# Fetal asphyctic preconditioning modulates the acute cytokine response thereby protecting against perinatal asphyxia in neonatal rats

**DOI:** 10.1186/1742-2094-10-14

**Published:** 2013-01-26

**Authors:** Evi Vlassaks, Eveline Strackx, Johan SH Vles, Maria Nikiforou, Pilar Martinez-Martinez, Boris W Kramer, Antonio WD Gavilanes

**Affiliations:** 1 Department of Pediatrics Division of Neonatology, Maastricht University Medical Center, School for Oncology and Developmental Biology Maastricht (GROW), Maastricht, AZ, 6202, The Netherlands; 2 Department of Neuropsychology Division Neuroscience, Maastricht University, School of Mental Health and Neuroscience (MHeNS), Maastricht, MD, 6200, The Netherlands; 3 Child Neurology, Maastricht University Medical Center, Maastricht, AZ, 6202, The Netherlands

**Keywords:** Asphyxia, Cytokines, Inflammation, Perinatal, Preconditioning

## Abstract

**Background:**

Perinatal asphyxia (PA) is a major cause of brain damage and neurodevelopmental impairment in infants. Recent investigations have shown that experimental sublethal fetal asphyxia (FA preconditioning) protects against a subsequent more severe asphyctic insult at birth. The molecular mechanisms of this protection have, however, not been elucidated. Evidence implicates that inflammatory cytokines play a protective role in the induction of ischemic tolerance in the adult brain. Accordingly, we hypothesize that FA preconditioning leads to changes in the fetal cytokine response, thereby protecting the newborn against a subsequent asphyctic insult.

**Methods:**

In rats, FA preconditioning was induced at embryonic day 17 by clamping the uterine vasculature for 30 min. At term birth, global PA was induced by placing the uterine horns, containing the pups, in a saline bath for 19 min. We assessed, at different time points after FA and PA, mRNA and protein expression of several cytokines and related receptor mRNA levels in total hemispheres of fetal and neonatal brains. Additionally, we measured pSTAT3/STAT3 levels to investigate cellular responses to these cytokine**s**.

**Results:**

Prenatally, FA induced acute downregulation in IL-1β, TNF-α and IL-10 mRNA levels. At 96 h post FA, IL-6 mRNA and IL-10 protein expression were increased in FA brains compared with controls. Two hours after birth, all proinflammatory cytokines and pSTAT3/STAT3 levels decreased in pups that experienced FA and/or PA. Interestingly, IL-10 and IL-6 mRNA levels increased after PA. When pups were FA preconditioned, however, IL-10 and IL-6 mRNA levels were comparable to those in controls.

**Conclusions:**

FA leads to prenatal changes in the neuroinflammatory response. This modulation of the cytokine response probably results in the protective inflammatory phenotype seen when combining FA and PA and may have significant implications for preventing post-asphyctic perinatal encephalopathy.

## Background

Perinatal hypoxia/ischemia or asphyxia (PA) is associated with a high mortality rate and causes multiorgan deficits including encephalopathy, leading to permanent structural brain damage [1,2]. Irrespective of the prevalence of PA, there is no effective treatment for term infants having post-asphyctic encephalopathy, except for hypothermia for moderate cases [3]. We have shown in a rat model that a brief episode of fetal asphyxia (FA) at embryonic day 17 (E17) lowers the severity of a subsequent more severe global asphyctic insult at term birth (FA preconditioning) [4]. FA preconditioned animals have less apoptosis in different regions of the brain and show improved locomotor function and object memory after PA compared with non-preconditioned animals [4,5]. The molecular mechanisms underlying FA preconditioning have however not been elucidated yet.

Several studies suggest that inflammation has a key role in perinatal brain injury, including cerebral hypoxia/ischemia [6,7]. Inflammatory cells, such as neutrophils and macrophages, but also resident brain cells, such as astrocytes and microglia, have been found to be activated by cerebral ischemia. These cells consequently produce inflammatory mediators, including reactive oxygen species and cytokines [8]. Human data revealed elevated interleukin (IL)-6 and tumor necrosis factor (TNF)-α levels in cerebrospinal fluid (CSF) from newborn infants with asphyctic encephalopathy [9]. Moreover, the concentrations of IL-6, IL-8 and IL-10 have been shown to be upregulated in the sera of asphyxiated neonates compared with those in normal neonates [10]. In experimental stroke models, where they studied the P7 carotid artery ligation model, they also found increased levels and activity of IL-1β, IL-6 and TNF-α several hours to days after the insult [11,12]. In contrast, Ashdown and coworkers reported lower IL-1β and TNF-α protein expression and lower IL-6 mRNA levels in neonatal rat brains 2 h after global PA [7]. Although special attention is needed concerning the controversies reported between focal and global asphyctic insults, all these experiments indicate that cytokines may modulate asphyctic damage in brain tissue. Moreover, inflammatory cytokines are implicated to be protective in ischemic brain tolerance [13-16]. It has been suggested that preconditioning ameliorates asphyxia-induced brain damage by suppressing the inflammatory response [13,16]. No experimental studies, however, have examined the role of cytokines after global FA preconditioning in a perinatal setting. Therefore, we aimed to investigate the inflammatory response that is activated during asphyctic brain damage. In addition, we investigated whether levels of pro- and antiinflammatory cytokines modulate neuroprotection against PA injury. To study this, we used a rat model where two global, instead of focal, asphyctic insults were combined to better reflect the pathology of global asphyctic encephalopathy and multiple organ failure in the human neonate [17,18]. We found that FA induces time-dependent cytokine changes, probably resulting in a protective inflammatory phenotype when followed by PA.

## Methods

### Animals and tissue processing

Animal experiments were approved and performed according to the guidelines of the Animal Ethics Board of Maastricht University, The Netherlands. Timed-pregnant Sprague–Dawley rats, obtained from Charles River (France), were housed individually under standard laboratory conditions. Pregnant rats were randomly assigned to an experimental group. Unsexed fetuses and male neonates were used in this study.

**Figure 1:**
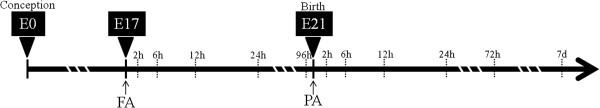
**Experimental design.** FA was induced at E17 by clamping the uterine vasculature for 30 min. At term birth, global PA was induced by placing the uterine horns containing the pups in a saline bath (37°C) for 19 min. All animals were delivered by Caesarean section. Pups were killed at five different time points after FA prenatally (*n* = 5 per group per time point) and six different time points postnatally (*n* = 4 per group per time point). E= embryonic day, FA= fetal asphyxia, PA= perinatal asphyxia.

For the collection of tissue samples, dams and pups were killed by decapitation at several time points after FA and at different time points after birth (*n* = 4–5) (Figure 1). Total brain hemispheres were collected from the offspring, snap-frozen in liquid nitrogen and preserved at −80°C for further analysis.

### FA preconditioning and PA

The current study used a rat model where two global asphyctic insults were combined. At E17, FA preconditioning was induced by performing a midline laparotomy in pregnant rats. Both uterine horns containing the pups were exposed, and the uterine and ovarian arteries were clamped using four removable clamps. After 30 min, the clamps were removed to allow reperfusion, the uterine horns were placed back intra-abdominally, and the abdominal cavity was closed. The procedures explained above were performed in a controlled environment at 37°C and 75% air humidity.

To assure full-term pregnancy and the physiology of labor, Caesarean section (C-section) was only performed after the vaginal delivery of the first-born pup. Control and FA pups were delivered immediately by C-section. To induce PA, pregnant rats were killed by decapitation to avoid the potential effect of the anesthetic. After hysterectomy, the uterine horns containing the pups were placed in saline (0.9% NaCl, 37°C) for exactly 19 min. Afterwards, pups exposed to the PA insult were delivered and stimulated manually to breathe in a closed incubator (37°C and 75% air humidity). The umbilical cords were ligated and cut to separate the pups from their placentas. Pups were randomly cross-fostered with surrogate dams (maximally 12 pups each dam) that had given birth vaginally on the same day.

### RNA isolation and RT-PCR

Total RNA was extracted from frozen brain tissue by homogenization of the samples with Trizol Reagent (Invitrogen, Breda, The Netherlands) according to the manufacturer’s guidelines. RIN values were determined using the Agilent 2100 Bioanalyzer (Agilent Technologies, Amstelveen, the Netherlands). All RNA samples had RIN > 8 and were included. Quantity of the RNA was determined with the Nanodrop (ND-1000 spectrophotometer; Thermo Scientific, Wilmington, DE, USA). Reverse transcription was carried out from 1 μg total RNA using the Revert Aid First Strand cDNA Synthesis Kit (Fermentas, St. Leon Rot, Germany) according to the manufacturer’s instructions. Then 5 μl of diluted cDNA (dilution 1:20) was amplified with LightCycler 480 SYBR Green I Master (Roche Applied Science, Almere, The Netherlands) in a final volume of 20 μl. The real-time PCR was performed on a LightCycler 480 system (Roche Applied Science; 45 cycles: 20 s at 95°C, 15 s at 60°C, 15 s at 72°C). Each PCR was carried out in duplicate, and samples negative for RevertAid Reverse Transcriptase were used as negative control. Investigated genes were: IL-1β and IL-1R1 and 2, IL-6 and IL-6R, TNF-α and TNFR1/p55 and 2/p75, and IL-10 and IL-10R (Table 1). To standardize for the amount of cDNA, cytokine expressions were compared to the geomean of β-actin, hypoxanthine-guanine phosphoribosyltransferase (HPRT) and glyceraldehyde 3-phosphate dehydrogenase (GAPDH) (Table 1). Quantification cycle values were extracted with the Lightcycler 480 software (Conversion LC and Linge PCR) and calculated based on the cycle threshold (Ct) values.

**Table 1:** Oligonucleotide primers used for RT-PCR

**Primer**	**Tm°**	**Sequence (5’-3’)**
HPRT for	64.1	TTGCTGGTGAAAAGGACCTC
HPRT rev	64.0	TCCACTTTCGCTGATGACAC
β-actin for	64.2	TTGCTGACAGGATGCAGAAG
β-actin rev	64.0	TGATCCACATCTGCTGGAAG
GAPDH for	63.4	CTCCCATTCTTCCACCTTTG
GAPDH rev	63.8	ATGTAGGCCATGAGGTCCAC
IL-1β for	67.0	TACCTATGTCTTGCCCGTGGAG
IL-1β rev	67.6	ATCATCCCACGAGTCACAGAGG
TNFα for	64.0	TGCCTCAGCCTCTTCTCATT
TNFα rev	63.2	GGGCTTGTCACTCGAGTTTT
IL-6 for	62.5	AAAGCCAGAGTCATTCAGAGC
IL-6 rev	63.8	GAGCATTGGAAGTTGGGGTA
IL-10 for	64.0	CCTGCTCTTACTGGCTGGAG
IL-10 rev	63.8	TGTCCAGCTGGTCCTTCTTT
IL-1R1 for	63.7	TTGTGTTTAAGCTGTTGCCG
IL-1R1 rev	62.4	GTAACCTCGATGGTATCTTCCC
IL-1R2 for	61.9	AGCACGTTTATCTCAGTGGC
IL-1R2 rev	65.5	GTGACTGGATCAAAAATCAGCG
TNFR1/p55 for	61.9	AGTCTACTGTGCCGATATCCC
TNFR1/p55 rev	64.9	CTTTGACAGGAGCTGAATCCC
TNFR2/p75 for	64.0	AAATGCAAGCACAGATGCAG
TNFR2/p75 rev	64.3	CAGCAGACCCAGAGTTGTCA
IL-6R for	63.8	CTGGTTCCTCTCCTCACCC
IL-6R rev	64.9	TGTTGCTGTTGTCATTAGGGC
IL-10R for	63.8	ATTTCACCGTGACCAACCTC
IL-10R rev	64.1	CCAGGATGTGAATGTCATCG

### Cytometric bead array

Pro- and antiinflammatory cytokine concentrations in brain homogenates were determined using the BD cytometric bead array (CBA) flex set kit (BD Biosciences, Breda, The Netherlands). The cytokine concentrations in tissue homogenates were calculated from standard curves generated using purified cytokines provided by the manufacturer. Brain pieces were homogenized using a Zymo Research bead beater for 2 × 20 s at 6 m/s in water yielding a 250 mg/ml tissue homogenate. The concentration of total protein was measured with the Bradford method. All samples were then incubated with microbeads for each of the cytokines. Analysis for IL-6, IL-10 and TNFα content was performed by flow cytometry according to the manufacturer’s directions. Analysis was performed using CBA software, which allows the calculation of cytokine concentrations in unknown samples [19].

### Immunohistochemistry

Brain hemispheres of postnatal pups were fixed in Somogyi, snap-frozen with CO_2_ and cut in 16-μm sagittal sections. Inflammatory cytokine expression was determined by immunohistochemical staining for IL-1β, TNF-α IL-6 and IL-10. After antigen retrieval, endogenous peroxidase activity was blocked by incubation in 3% hydrogen peroxide. Nonspecific binding sites were blocked with serum. Cytokines were stained with rabbit polyclonal anti-IL1β (Santa Cruz Biotechnology, dilution 1:50), goat polyclonal anti-TNF α (Santa Cruz Biotechnology, dilution 1:200), goat polyclonal anti-IL6 (R&D Systems, dilution 1:200) and goat polyclonal anti-IL-10 (Santa Cruz Biotechnology, dilution 1:100). Biotinylated polyclonal swine anti-rabbit IgG and anti-goat (DAKO, dilution 1:200/1:500) were applied as secondary antibody. Staining was performed with DAB, and sections were counterstained with cresyl violet. Sections were photographed using a Nikon digital camera DMX1200 and ACT-1 v2.63 software from Nikon Corporation.

### Western blotting

Frozen brain tissue was homogenized in CelLytic MT Mammalian Tissue Lysis/Extraction Reagent (Sigma, St-Louis, MO, USA). The samples were then centrifuged at 15,000 g for 10 min. The protein-containing supernatants were collected, and the concentration of total protein was measured with the Bradford method. Proteins were denatured by heating at 95°C for 10 min. Equal amounts of protein (30μg) from each sample were loaded onto acrylamide gels and separated by SDS-PAGE, and protein from the gel was transferred onto a nitrocellulose membrane. Membranes were incubated overnight at 4°C with rabbit anti-pSTAT3-Tyr705 and rabbit anti-STAT3 (dilution 1:1,000; Cell Signaling). Monoclonal mouse anti-rabbit GAPDH (dilution 1:2,000,000, Fitzgerald Industries) was used as a loading control. The blots were analyzed using the LICOR Odyssey Infrared Imaging System, and images were acquired using Adobe Photoshop CS4 software.

### Statistical analysis

Statistical analysis was performed with Statistical Package for Social Sciences (SPSS 17.0 Software). Prenatal data were analyzed using the Mann–Whitney test. All postnatal data were analyzed using the one-way analysis of variance (ANOVA) test, followed by post-hoc comparisons using LSD correction. Multivariate testing was performed to test the interaction between birth and asphyxia. Results are presented as means + SEM and *p*-values ≤ 0.05 were considered statistically significant.

## Results

The impact on the cytokine expression of FA prenatally and in combination with a subsequent PA insult postnatally was assessed over a longitudinal time pattern until P7 (Figure 1). Both FA and PA induced acute cytokine changes in the brain. From P3 onwards, no significant differences were found in mRNA levels for any of the cytokines or receptors compared with control levels (data not shown).

### Acute downregulation of IL-1β, TNF-α and IL-10 mRNA levels but increased IL-6 mRNA and IL-10 protein expression occurred 96 h after fetal asphyxia

**Figure 2:**
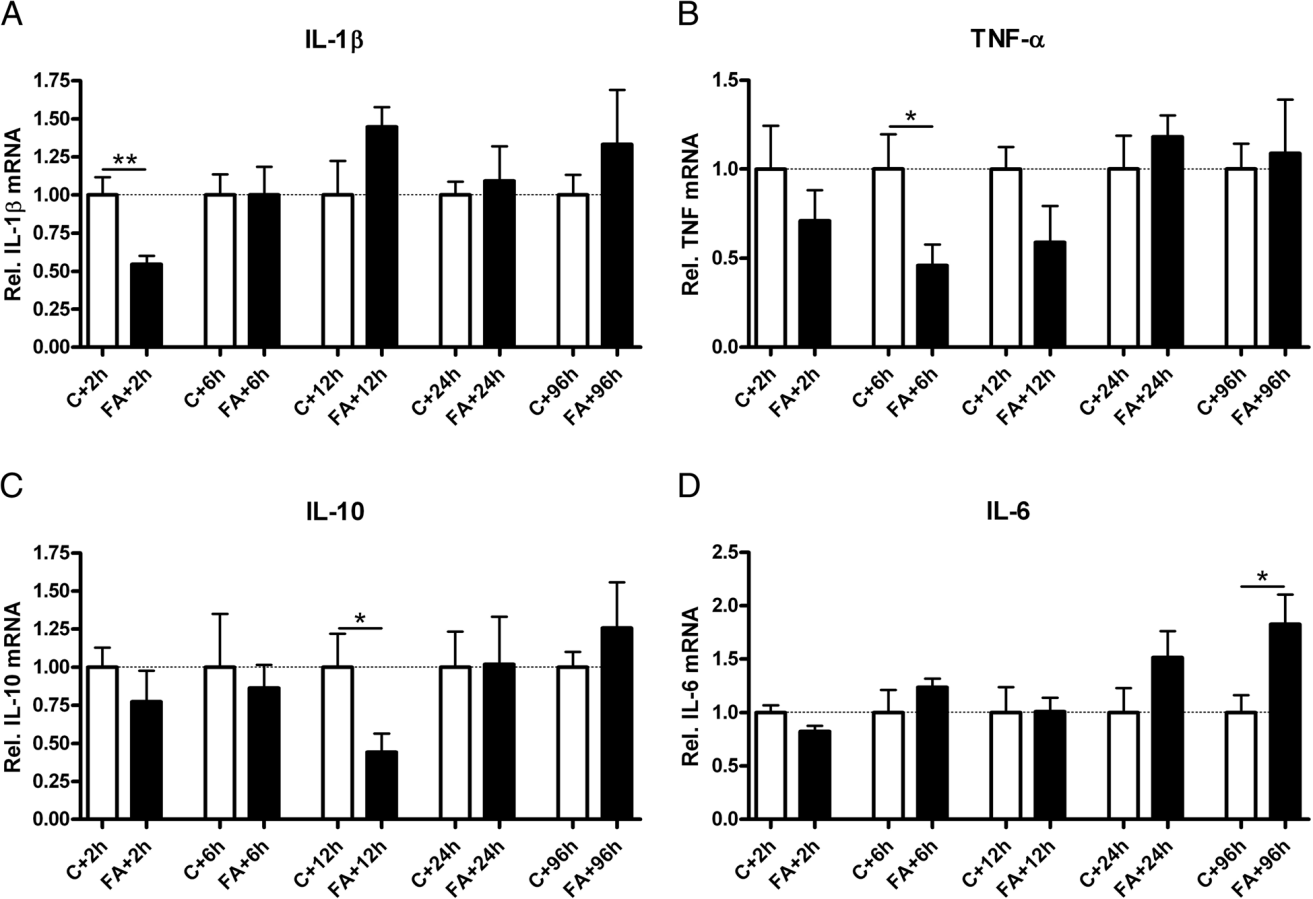
**Acute downregulation of IL-1β, TNF-α and IL-10 mRNA levels but increased IL-6 mRNA levels after fetal asphyxia.** Prenatal relative mRNA levels of IL-1β (**A**), TNF-α (**B**), IL-10 (**C**) and IL-6 (**D**) in control and FA animals. mRNA levels are relative to the geomean of measured housekeeping genes and normalized to control levels. *p < 0.05 and **p < 0.01 significantly different from respective control group. Data shown as mean + SEM.

Prenatally, FA induced an acute downregulation of IL-1β at 2 h (Figure 2A; *p* = 0.008) and TNF-α at 6 h (Figure 2B; *p* = 0.05) compared with respective controls. The antiinflammatory cytokine IL-10 followed the same pattern as the acute inflammatory response of IL-1β and TNF-α, with decreased IL-10 mRNA levels at 12 h after FA (Figure 2C; *p* = 0.02). In contrast to the decreased levels of the other cytokines measured in this study, IL-6 mRNA levels were elevated 96 h after FA compared with controls (Figure 2D; *p* = 0.03). All receptors for indicated cytokines showed mainly an elevation in mRNA levels after FA, mostly at time points after their respective cytokine response (data not shown).

**Figure 3:**
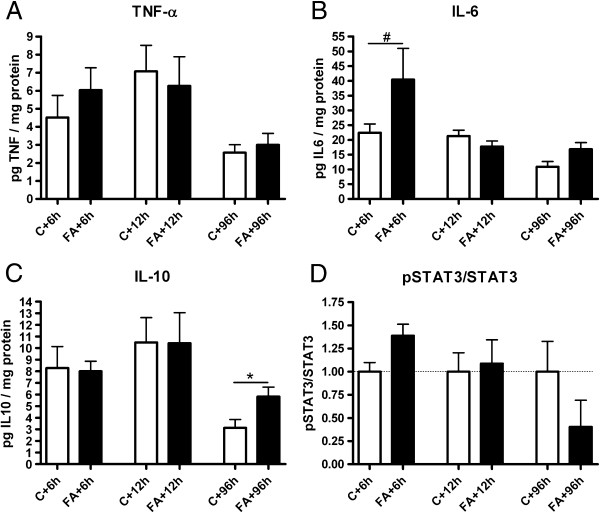
**Increased IL-10 protein expression at 96 h post FA**. Prenatal protein concentrations of TNF-α (**A**), IL-6 (**B**) and IL-10 (**C**) expressed in pg/mg protein in control and FA animals. Relative protein expression of pSTAT3/STAT3 normalized to GAPDH and to respective control levels (**D**). # 0.08<*p* <0.05; *p < 0.05 significantly different from respective control group. Data shown as mean + SEM.

Protein expression of TNF-α, IL-10 and IL-6 was determined at 6, 12 and 96 h after FA. CBA analysis revealed increased IL-6 levels at 6 h post FA, although this was not significant (Figure 3B; *p* = 0.08). IL-10 levels, however, were significantly increased at 96 h after FA compared with control levels (Figure 3C; *p* = 0.04).

Since phosphorylated signal transducer and activator of transcription (STAT)-3 plays a role in post-ischemic brain damage [20] and all the cytokines that we measured can mediate their effects through pSTAT3 signaling, we measured the amount of pSTAT3 in control and FA brains. We could not find any significant changes in pSTAT3/STAT3 levels at 6, 12 or 96 h post FA (Figure 3D).

### An asphyctic insult acutely downregulates the proinflammatory response 2 h after birth

**Figure 4:**
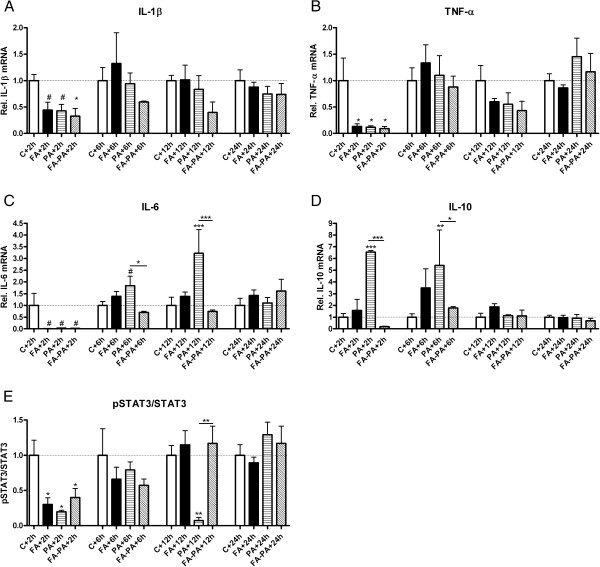
**Acute downregulation of the proinflammatory response 2 h after birth in all experimental groups.** Increased IL-10 and IL-6 mRNA levels in non-preconditioned PA animals, but baseline IL-10 and IL-6 mRNA levels in preconditioned animals. Postnatal relative mRNA levels of IL-1β (**A**), TNF-α (**B**), IL-6 (**C**) and IL-10 (**D**) in control, FA, PA and FA-PA animals. mRNA levels are relative to the geomean of measured housekeeping genes and normalized to control levels. Relative protein expression of pSTAT3/STAT3 normalized to GAPDH and to respective control levels (**E**). # 0.08<*p* < 0.05; *p<0.05 and **p < 0.01 indicate significant differences between groups. Data shown as mean + SEM.

Postnatally, IL-1β, TNF-α and IL-6 mRNA levels decreased 2 h after birth in all experimental groups compared with control levels (Figure 4A-C).

**Figure 5:**
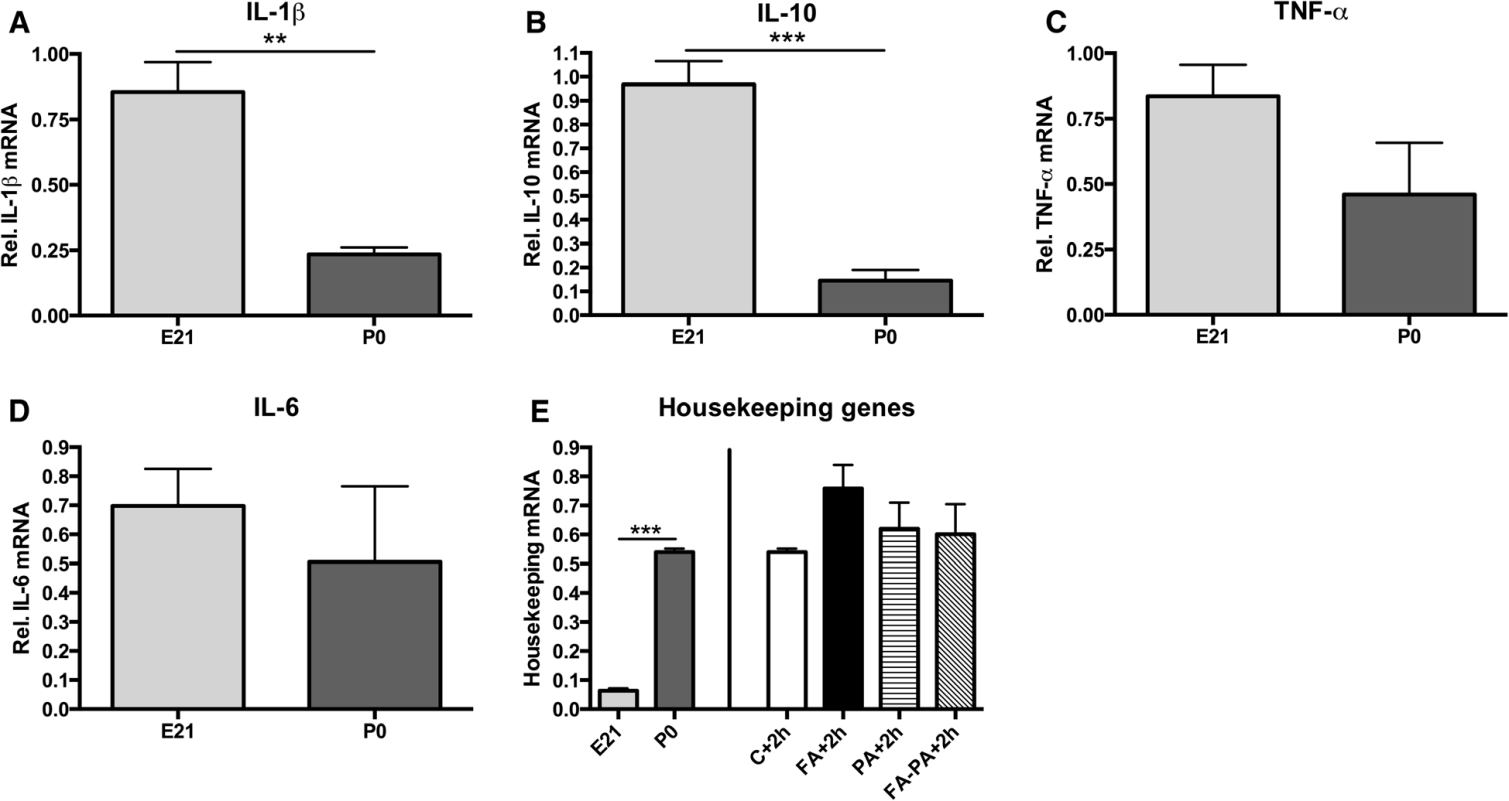
**Comparison between E21 and P0 cytokine response.** mRNA levels of IL-1β (**A**), IL-10 (**B**), TNF-α (**C**) and IL-6 (**D**) are presented relative to the geomean of measured housekeeping genes. mRNA levels of housekeeping genes from control animals at E21 and P0 (left panel) and housekeeping mRNA levels at P0 in all groups (right panel) (**E**). **p < 0.01 and ***p < 0.001 indicate significant differences between groups. Data shown as mean + SEM.

To investigate if the decrease in proinflammatory cytokines 2 h after birth was attributable to the effect of birth itself or if it was specifically due to asphyxia, we compared cytokine levels from control E21 animals with control P0 animals (2 h after birth). Comparisons revealed that IL-1β and IL-10 decreased because of birth (Figure 5A and B; *p* < 0.01 and *p* < 0.001, respectively). mRNA levels for TNF-α and IL-6, however, did not differ between E21 and P0 animals [(Figure 5C and D); *p* = 0.12 and *p* = 0.47, respectively)]. Hence, we studied the mRNA levels of the housekeeping genes to assess if the decrease in IL-1β and IL-10 was attributable to a more general downregulation of the cellular response. Figure 5E shows that the housekeeping genes increased 2 h after birth in comparison with the E21 animals (left panel; *p* < 0.001). Moreover, the housekeeping genes were not affected by an asphyctic insult (Figure 5E, right panel). These results indicate that IL-1β and IL-10 decrease because of the birth process and that this response is specific for these cytokines. However, because IL-1β levels were even lower in animals that experienced an asphyctic insult, we assessed whether a synergic effect between birth and asphyxia was present. Surprisingly, multivariate testing between prenatal (E21) and postnatal (P0, 2 h after birth) animals revealed that the birth process interacts with the asphyctic insult for TNFα, IL-6 and IL-10 (*p* < 0.05) but not for IL-1β.

Although no changes in protein expression could be observed 2 h after birth (data not shown), the downregulation in cytokine mRNA levels did lead to decreased levels in pSTAT3/STAT3 2 h after birth in all experimental groups (Figure 4E; *p* < 0.05).

### IL-10 and IL-6 mRNA levels are increased after perinatal asphyxia but attenuated in FA preconditioned animals

Postnatally, IL-10 dissociated from the observed proinflammatory response with increased mRNA levels 2 h and 6 h after the PA insult. This effect was attenuated in the preconditioned animals (FA-PA group) (Figure 4D; *p* < 0.001). Interestingly, at 6 h and 12 h after birth an IL-6 mRNA profile similar to that for IL-10 was observed. More specifically, at 6 h IL-6 mRNA levels were reaching almost statistical significance in animals previously exposed to PA (Figure 4C; *p* = 0.06). Animals that were also exposed to the FA preconditioning stimulus (FA-PA group), however, showed baseline IL-6 mRNA levels (Figure 4C; *p* = 0.05 for PA vs. FA-PA). This effect was expressed even more strongly at 12 h with increased IL-6 mRNA in PA animals (Figure 4C; *p* < 0.001) and baseline IL-6 mRNA levels in preconditioned animals (Figure 4C; *p* < 0.001 for PA vs. FA-PA).

Despite the higher mRNA levels for IL-10 and IL-6 after PA, we could not find any significant changes in protein expression (data not shown). Surprisingly, at 12 h after PA the pSTAT3/STAT3 levels were decreased (*p* < 0.01), while FA-PA animals revealed comparable levels as control animals (*p* < 0.01; Figure 4E).

### Diverse localization of measured cytokines in P7 brains

**Figure 6:**
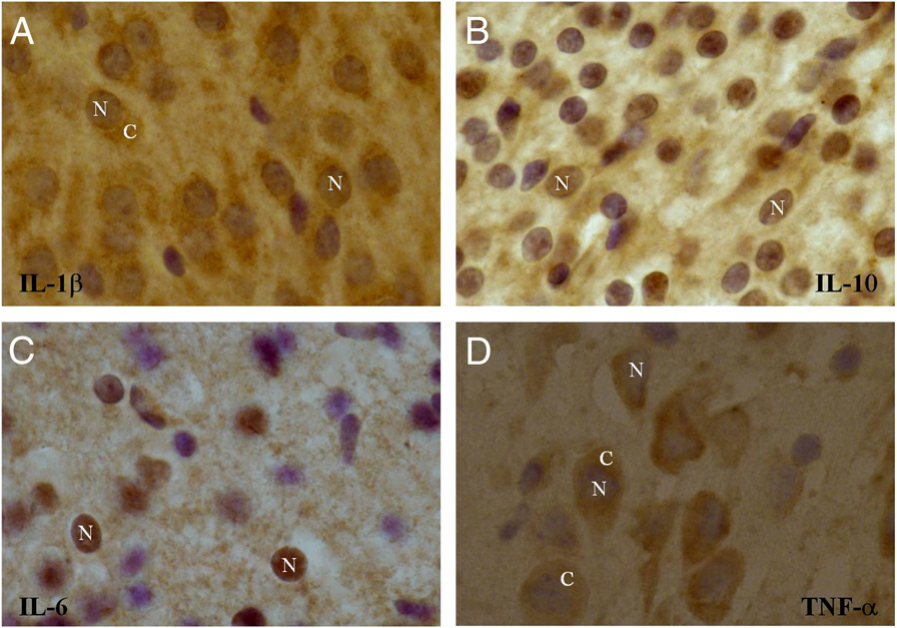
**Diverse localization of measured cytokines in P7 brains**. Representative pictures (100× magnification) of immunohistochemical stainings of IL-1β (**Α**), IL-10 (**B**), IL-6 (**C**) and TNF-α (**D**). Cytokines are stained with DAB (*brown color*) and counterstained with cresyl violet.

Immunohistochemical stainings of IL-10, IL-6, IL-1β and TNF-α were performed to localize the cytokines in P7 rat brains. We observed that IL-1β is expressed in both the nucleus and cytosol (Figure 6A). IL-10 and IL-6 are mainly expressed in the nucleus (Figure 6B-C), while TNF-α is localized in the cytosol (Figure 6D). All cytokines also seemed to be expressed extracellular.

## Discussion

In this study, we aimed to elucidate the inflammatory cytokine profile in response to global fetal and perinatal asphyxia. Moreover, we investigated whether pro- and antiinflammatory cytokines played a role in the fetal induction of endogenous neuroprotection against PA injury. Prenatally, a time-dependent cytokine profile was observed after FA with acute downregulation of IL-1β, TNF-α and IL-10 mRNA levels. At 96 h post FA, immediately before birth, IL-6 mRNA and IL-10 protein expression were increased. Postnatally, PA rats showed higher IL-10 and IL-6 mRNA levels after PA than control rats. Moreover, in FA preconditioned rats this effect was attenuated, showing an inflammatory phenotype similar to control animals.

Cytokines have been demonstrated to play an important role in the brain response to asphyxia. Moreover, they have been implicated in playing a protective role in the induction of ischemic tolerance [13,16]. The exact role of these cytokines and the time pattern of their expression have, however, not been elucidated yet. In human studies, IL-1β, IL-6 and TNF-α have been found to be upregulated in CSF of asphyxiated newborns compared with control neonates [9,21]. In these studies, CSF sampling was performed at only one time point (24 h and 48 h respectively) after birth. More time points are included in experimental models of focal asphyxia. These models are mainly based on the Rice-Vannucci model [22], where P7 rats are exposed to unilateral carotid artery occlusion, followed by a period of systemic hypoxia. Although these models are widely used to investigate the mechanisms involved in PA, they miss the transitional physiology from intrauterine to extrauterine life. The birth process itself is a physiologically unique period, with specific hormonal changes priming the fetus to be born [23]. This aspect was covered by the model used in our study as we awaited the vaginal delivery of the first pup so that the physiological conditions that occur during labor would be mimicked.

One aim of our study was to investigate the inflammatory mechanisms in global asphyxia. To the best of our knowledge, the role of cytokines in preconditioning during the fetal period has not been studied before. Therefore, we explored the impact of 30-min sublethal global fetal asphyxia on the levels of IL-1β, TNF-α, IL-10 and IL-6.

A time-dependent cytokine response after FA was observed prenatally. This response started 2 h after FA with decreased IL-1β mRNA levels, followed by a drop in TNF-α mRNA levels at 6 h post FA. Both IL-1β and TNF-α are key mediators of the acute inflammatory response [24] and have been shown to be upregulated after global cerebral hypoxia-ischemia in adult and neonatal rats [12,25,26]. Important differences between the addressed studies and our observations include the nature and the time point of the asphyctic insult. The FA that we induced is only a sublethal insult with no fetal losses or morbidity in adulthood [4]. Additionally, we induced the stimulus prenatally, and it has been suggested that the fetal brain is less reactive to inflammatory reactions than a mature brain [26].

The antiinflammatory cytokine also showed decreased mRNA levels at 12 h after FA. IL-10 is an important antiinflammatory mediator of the immune response and has been shown to be both upregulated [27] and downregulated [28] after an hypoxic-ischemic insult. In addition, IL-10 prevents neurodegeneration by protecting neurons through activation of PI-3-kinase and STAT-3 pathways [29,30]. Interestingly, at 96 h after FA protein expression of IL-10 was increased together with increased IL-6 mRNA levels. It is known that IL-6 is secreted together with the antiinflammatory cytokine IL-10 [21,31], and IL-6 is a pleiotropic cytokine with both pro- and antiinflammatory effects in the injured brain [32,33]. IL-6 has been shown to be expressed more on neurons in response to ischemia, promoting neuronal survival and development [34]. Furthermore, a study by Yamashita and coworkers showed that endogenous IL-6 plays a critical role in preventing apoptosis of damaged neurons in the acute phase of cerebral ischemia in mice [35].

Taken together, these data indicate that FA induces acute changes in the cytokine response. Since IL-6 mRNA and IL-10 protein levels are increased at 96 h post FA, the time point immediately before birth, these cytokines might be involved in inducing neuroprotection.

The second aim of our study was to investigate whether the changes in cytokine response observed because of FA have any protective effect against a subsequent more severe PA insult. Our data showed decreased mRNA levels of the proinflammatory cytokines (IL-1β, TNF-α and IL-6) 2 h after birth in all experimental groups compared with control animals. Moreover, pSTAT3/STAT3 levels were decreased, indicating a downregulation in proinflammatory response. This decrease in cytokine levels is in accordance with a study from Ashdown et al., who observed decreased IL-1β and TNF-α protein and decreased IL-6 mRNA in the brain at 2 h after birth asphyxia [7]. They claimed that the downregulation in cytokine levels was due to global birth asphyxia itself and not to the C-section. Comparison of E21 and P0 control animals revealed that IL-1β and IL-10 levels decreased because of birth. Control levels of TNF-α and IL-6 did not show decreased levels due to birth. These are remarkable findings because studies have shown that the proinflammatory response is increased during the birth process [36]. Additionally, although IL-1β control levels decreased 2 h after birth, these levels were lower in the experimental groups than in the control groups, suggesting an interaction between the birth process and asphyxia. However, a synergic effect between birth and any asphyctic insult (FA and/or PA) was not observed for IL-1β but for the other cytokines. Hence, the drop in inflammatory cytokines 2 h after birth remains an interesting and important observation because focal asphyctic episodes have been associated with increased inflammation [6,11,12,26]. Studies indicate that endogenous production of glucocorticoids may play an important role in the suppression of specific cytokine production and inflammation [37]. As it has been established that birth is associated with higher stress hormones, such as cortisol and catecholamines [38], and hypoxia induces an even higher catecholamine response in the fetus [39], we can assume that the animals that had experienced an asphyctic insult before had higher corticosteroid levels. Accordingly, these animals would be able to downregulate the proinflammatory response as a protective mechanism.

One of the main findings of our study is that IL-10 was highly expressed in asphyxiated animals 2 h after birth. Moreover, significantly elevated mRNA levels of IL-6 were observed at 6 h and 12 h after PA. These data are comparable to the abundance of evidence indicating increased cytokine levels in the brain due to hypoxia-ischemia [6,11,12,26]. Most importantly, a preconditioning effect was observed in both IL-10 and IL-6 mRNA levels. So the increase in IL-10 and IL-6 mRNA caused by PA was attenuated and comparable to control levels in the preconditioned FA-PA animals. Studies have already demonstrated that inhibition of cytokine activity reduces excitotoxic brain injury in neonatal rats [11,40,41]. An important role for the cytokines IL-10 and IL-6 after global asphyxia is highlighted in our study. An IL-6 response to asphyxia has been correlated with higher mortality rates [21]. Therefore, the changes in IL-6 levels could indicate why FA preconditioned animals have a higher survival chance after PA compared with non-preconditioned animals. Although both human and experimental data show increased levels of IL-10 and IL-6 after asphyctic injury [9,11,12,28,31], the precise role of these cytokines in cerebral asphyxia has not been fully elucidated. Hence, further studies on the role of IL-10 and IL-6 in the pathophysiology of global perinatal brain injury are warranted.

It has been shown that the post-ischemic inflammatory neuronal damage is amplified by the STAT signaling pathways [42,43]. Additionally, prevention of the post-ischemic STAT3 phosphorylation leads to significant neuroprotection and a decrease in neurological deficits [20]. Surprisingly, we found decreased pSTAT3 expression 12 h post PA, while preconditioned animals showed baseline pSTAT3 expression. We can assume that the downregulation in pSTAT3 levels is an attempt to promote neuronal survival since we are looking at the brains of surviving offspring.

## Conclusions

In this study, we report important findings concerning the inflammatory mechanisms of global asphyctic brain damage and the role of inflammatory cytokines in the induction of neuroprotection against perinatal asphyctic injury. Consistent with our hypothesis, we demonstrated that FA induces time-dependent cytokine changes with acute downregulation of IL-1β, TNF-α and IL-10 mRNA, followed by increased IL-6 mRNA and IL-10 protein expression. Importantly, these prenatal changes downregulate the cytokine response when combined with a global asphyctic insult at birth. Although we did not observe any changes in cytokine levels from 24 h after PA, we previously showed that PA induces astrogliosis and apoptosis at 7 days post PA, which will lead to motor and memory deficits in adulthood [4]. Therefore, gaining more insights into the inflammatory pathways of IL-6 and IL-10 acutely after global asphyxia may have significant implications for preventing or modulating the permanent effects of post-asphyctic encephalopathy.

## Competing interests

There are no competing interests for this manuscript.

## Authors’ contributions

EV performed all animal work, cytokine mRNA/protein measurements, Western blots and stainings, performed statistical analysis and wrote the manuscript. ES assisted with the animal work and edited the paper. MN assisted with the animal work and all cytokine measurements. JSHV, PM, BWK and AWDG provided critical guidance and worked on the manuscript. All authors read and approved the final manuscript.
